# Evaluation of participation and performance indicators in a breast cancer screening program in Saudi Arabia

**DOI:** 10.15537/smj.2022.43.11.20220269

**Published:** 2022-11

**Authors:** Yasser M. Alatawi, Hala A. Alshomrani, Sara M. Baeshen, Hayat H. Alkhamisi, Roaa M. Almazrui, Mohammed S. Alghamdi, Sara M. Bugshan, Tarik K. Alafif, Hussam A. Hijazi, Jawaher R. Alahmadi, Sawsan A. Ashoor, Ahmad M. Alamri, Faris Alkhilaiwi

**Affiliations:** *From the Department of Pharmacy Practice (Alatawi), Faculty of Pharmacy, University of Tabuk, Tabuk; from the Department of Natural Products and Alternative Medicine (Alshomrani, Baeshen, Alkhamisi, Almazrui, Alghamdi, Alkhilaiwi), Faculty of Pharmacy, King Abdulaziz University; from the Sheikh Mohammed Hussein Al-Amoudi Center of Excellence in Breast Cancer (Bugshan), King Abdulaziz University; from the Department of Radiology (Hijazi, Alahmadi, Ashoor), Faculty of Medicine, King Abdulaziz University Hospital; from the Regenerative Medicine Unit (Alkhilaiwi), King Fahd Medical Research Center; King Abdulaziz University, Jeddah; from the Computer Science Department (Alafif), Jamoum University College, Umm Al-Qura University, Jamoum; from the Department of Clinical Laboratory Sciences (Alamri), College of Applied Medical Sciences, King Khalid University; from the Cancer Research Unit (Alamri), King Khalid University, Abha, Kingdom of Saudi Arabia.*

**Keywords:** breast cancer, cancer screening, mammography, quality indicators

## Abstract

**Objectives:**

To evaluate early performance indicators for breast cancer screening at the King Abdulaziz University Hospital in Saudi Arabia.

**Methods:**

This study retrospectively evaluated data from women who underwent their first breast cancer screening program in Jeddah, Saudi Arabia between 2012 and 2019. Data on screening results were used to estimate performance indicators and generate descriptive statistics.

**Results:**

Of the 16000 women invited from 2012 to 2019, a total of 1911 (11.9%) participated. The majority of women (68.8%) were between 40 and 55 years old. Based on the screening process results, 26.6%, 40.1%, 9.7%, 1.3%, 0.7%, and 5.2% of women had BI-RADS scores of R1, R2, R3, R4, R5, and R0 respectively. The remaining 16.3% did not have mammogram records. The recall rate, or the percentage of women who underwent further evaluation, was 19.9%; 18.9% underwent a biopsy procedure. In addition, 1.6% of women had cancer screen-detected, although only 0.7% were diagnosed with breast cancer.

**Conclusion:**

In light of the low participation and high recall rates, it is essential that the screening program utilizes performance indicators to optimize resource utilization and ensure the quality of the service provided. Additionally, a national framework and standardized performance indicators could mitigate this problem for other cancer screening programs.

**B**reast cancer is a leading cause of death worldwide and a serious public health issue that affects many people.^1^ Breast cancer is the most common form of cancer in Saudi Arabia, according to a recent systematic review.^1^ In 2020, 14.2% of new cancer diagnoses in Saudi Arabia were breast cancer, and the breast cancer mortality rate was 8.4%.^2^ Early detection of breast cancer results in a 98.8% increase in cure probability and a nearly 40% reduction in mortality.^3-5^ However, more than half of breast cancers in Saudi Arabia are detected at an advanced stage, compared to 20% in more advanced countries.^6^

Early detection of breast cancer involves 3 processes: mammography, breast self-examination, and clinical breast examination.^6,7^ According to a large meta-analysis, mammography screening programs reduce breast cancer mortality by 33%.^8^ Additionally, mammography programs can minimize late-stage cases, as seen in many industrialized countries.^7,9,10^ Therefore, many countries have developed breast cancer screening mammography programs.^8^ Although mammography can be part of an effective screening program in Saudi Arabia, the culture’s traditional values still limit its efficacy.^11-13^

As part of its commitment to achieving Vision 2030, Saudi Arabia launched the National Transformation Program in 2016. The new Transformation and Health Care Model consists of actions targeted toward preventing disease, including breast cancer.^14^ This notion requires the availability of data regarding the performance of breast cancer screening programs in the country. Moreover, monitoring early indicators of breast cancer screening can help optimize resource utilization and improve the quality of service.^6,15,16^

The purpose of this study is to examine early performance indicators for the cancer screening program at King Abdulaziz University Hospital in Saudi Arabia.

## Methods

**Figure 1:**
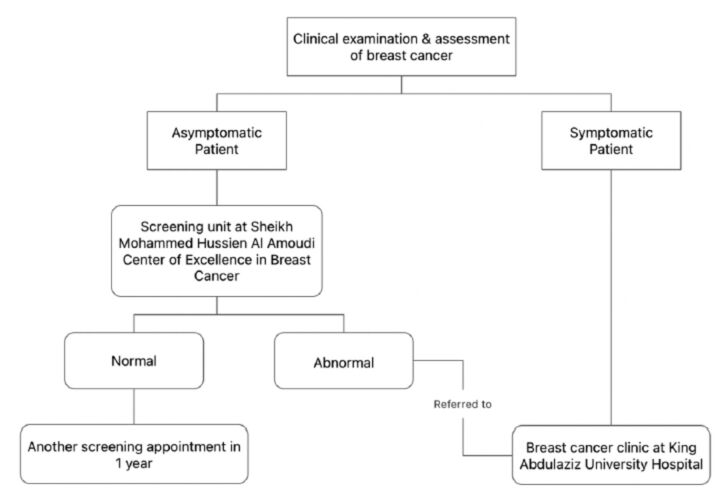
- The flowchart shows the breast cancer screening process at Sheikh Mohammed Hussien Al Amoudi Center of Excellence in the Breast Cancer Screening Unit at King Abdulaziz University, Jeddah, Kingdom of Saudi Arabia.

A retrospective analysis of cancer screening data from 2012 to 2019 was carried out in Jeddah, Saudi Arabia. The Breast Cancer Screening Center of Excellence was established at King Abdulaziz University Hospital in Jeddah in 2010; an overview of the screening process can be found in Figure 1. The program aimed to reach women 35-60 years old with a family history of breast cancer residing in Jeddah. The analysis included all participants whose screening records had been completed. Study approval was obtained from the Research Committee and the Institutional Review Board of the King Abdulaziz University) protocol code 54420 and approval date of November 2020), ensuring conformity with the Declaration of Helsinki.

The data were collected based on the information provided by the cancer screening center. There were 3 main categories of data. The first included information on demographics and risk factors, such as age, marital status, education, menopausal status, late parity, hormone replacement therapy, previous breast cancer history, and a family history of breast cancer. The second included detailed information regarding mammography examination readings taken by 2 oncologists. The Breast Imaging-Reporting and Data System (BI-RADS) of the American College of Radiology was used to score mammograms. The third included information on the mammogram results, including whether further procedures or imaging were required.

### Statistical analysis

Performance indicators were estimated based on the screening data. Indicators were chosen based on extensive discussions with the center’s oncologists, data availability, and literature review.^17^ Performance indicators included participation rate, recall rate, diagnostic workup rate, biopsy rate, detection rate, episode sensitivity, and percentage of confirmed cases. Each of these performance indicators is defined in Table 1. The descriptive statistics were calculated using SAS statistical software (SAS Institute, Inc., Cary, NC, USA).

**Table 1:** - Definition of performance indicators of the breast cancer screening program.

Indicators	Definition
Participation rate	Number of screened women divided by the number of invited women
Recallrate	Number of women who undergo further assessment for medical reasons based on a positive screening examination divided by the number of women screened
Rate of diagnostic workups: Ultrasound alone	Number of women who undergo ultrasound assessment divided by the number of women screened
Rate of diagnostic workups: Ultrasound with mammography	Number of women who undergo an ultrasound and mammogram assessment divided by the number of women screened
Biopsy rate	Number of women recalled for biopsy divided by the number of women screened
Detection rate	Number of women who were cancer screen-detected divided by the number of women screened
Percent of confirmed breast cancer diagnosis	Number of confirmed cancer cases divided by the number of women screened
Episode sensitivity	Number of women screen-detected for cancer divided by the number of all cancers detected

## Results

There were 1911 women enrolled in the screening program between 2012 and 2019. The demographics of the screening program participants are presented in Table 2. Most of the women (68.8%) were between the ages of 40 and 55 years. Approximately half of the women received hormonal replacement therapy. In addition, 19.8% had a family history of breast cancer. Only 6 women reported a history of breast cancer, and 55 reported a history of benign breast tumors. Eighty-nine women (4.7%) reported having undergone breast surgery (Table 3).

**Table 2:** - Characteristics of women enrolled in the program.

Variables	n (%)*
***Age group***	
<40	121 (6.3)
40-45	435 (22.8)
46-50	468 (24.5)
51-55	412 (21.6)
56-60	255 (13.3)
61-65	143 (7.5)
>60	67 (3.5)
No data	10 (0.5)
***Marital status***	
Married	1255 (65.7)
Single	178 (9.3)
Other/no data	478 (25.0)
***Education level***	
No education	50 (2.6)
Secondary school	197 (10.3)
High school	333 (17.4)
College degree	750 (39.2)
No data	581 (30.4)
***Parity***	
Yes	1873 (98.0)
No	38 (2.0)
***Nationality***	
Saudi	950 (49.7)
Non-Saudi	961 (50.3)
***Estrogen use***	
Yes	953 (49.9)
No	958 (50.1)
***Early menarche***	
Yes	145 (7.6)
No/no data	1766 (92.4)

*Percentages might not add to a 100 due to rounding

**Table 3:** - Risk factors and history of surgery of breast cancer

Risk factors*	n (%)
Family history of breast cancer	
Yes	379 (19.8)
No	1532 (80.2)
Mother (Yes)	110 (5.8)
Sister (Yes)	98 (5.1)
Daughter (Yes)	10 (0.5)
Other relative (Yes)	193 (10.1)
History of breast cancer (Yes)	6 (0.31)
History of benign breast diseases (Yes)	55 (2.9)
History of ovarian cancer (Yes)	6 (0.3)
History colon cancer (Yes)	5 (0.3)
History of cervical cancer (Yes)	10 (0.5)
History of other cancer (Yes)	24 (1.3)
History of surgery	
Lumpectomy (Yes)	32 (1.7)
Mastectomy (Yes)	6 (0.3)
Breast oncoplastic surgery (Yes)	36 (1.9)
Implants (Yes)	15 (0.8)

*Participants might be counted more than once if they have multiple risk factors.

The following distribution of BI-RADS scores was observed among the enrolled women: 26.6% had a score of R1, 40.1% had a score of R2, 9.7% had a score of R3, 1.3% had a score of R4, 0.7% had a score of R5, 5.2% had a score of R0, and 16.3% did not have mammogram records. We further classified patients into 3 categories based on the BI-RADS score. Scores of R1 and R2 indicated no further assessment or imaging was necessary. Scores of R3 and R0 indicated further assessment or imaging was necessary. Scores of R4 and R5 indicated suspected malignancy requiring additional imaging. Among the 1,275 women in the BI-RADS R1 and R2 groups, 9.5% had follow-up imaging, and only 0.2% had follow-up procedures. Of the 286 women in the BI-RADS R3 and R0 groups, 72.7% had follow-up imaging, and only 1.4% had follow-up procedures. A small proportion of women in the program were in the BI-RADS R4 and R5 groups (n=38); 55.3% received follow-up imaging, and 50% underwent follow-up procedures.

With regard to the performance indicators of the screening program, 11.9% of the 16,000 invited women participated between 2012 and 2019. The recall rate, or the proportion of women who underwent further assessment, was 19.9%; 18.9% underwent a biopsy procedure. Of the participants, 1.6% were cancer screen-detected, but only 0.7% were diagnosed with breast cancer. The episode sensitivity, which was defined as the number of women who were screen-detected for cancer divided by the number of confirmed cancers, was 2.4% (Table 4).

**Table 4:** - Performance indicators of the breast cancer screening program.

Indicators	Percent
Participation rate	11.9
Recall rate	19.9
Rate of diagnostic workups: ultrasound alone	19.9
Rate of diagnostic workups: ultrasound with mammography	13.3
Biopsy rate	18.9
Detection rate	1.6
Percent of confirmed breast cancer diagnosis	0.7
Episode sensitivity	2.4

## Discussion

This study aimed to evaluate a breast cancer screening program and its performance indicators. Sixteen thousand women were invited between 2012 and 2019. The participation rate was 11.9%, higher than the 7.8% participation rate reported in Kuwait.^18^ In contrast, this participation rate was much lower than those in developed countries such as Denmark (83.5%) and Germany (56.3%).^19^ The low participation rate may be explained by a lack of knowledge and sociocultural factors.^12,20^ For example, a previous study in Saudi Arabia found that women had sufficient knowledge regarding breast cancer risk factors and symptoms, but little information on proper screening methods was available.^20^

We observed a recall rate of 19.9%, which is higher than the recall rate of 8.8% reported in a large systematic review.^21^ Furthermore, the recall rates range from 2.6% to 4.6% in Denmark and Germany.^19^ We also found that 18.9% of the women had undergone a biopsy, which was higher than the 3.9% rate previously reported.^21^ For screening mammography, the Agency for Healthcare Research and Quality recommends a recall rate of 10%, as evidence suggests that a high recall rate results in fewer cancer diagnoses and more false positives.^18,22,23^ The annual reading volume and radiologists’ experience may have been related to the screening’s high recall rate.^24^ A system that targets radiologist factors (for example, screening volumes and second reviews of potential recalls) may reduce unwarranted high screening recalls.^25^

### Study limitations

There were several limitations to this study, including the fact that we had to rely on only one program, which limited the generalizability of the results. Additionally, the current study evaluated the recorded data of the program participants; the researchers had no control over the quality of the data collection. Several cases contained missing data, although this did not affect the study’s findings. Using a prospective method for collecting data will allow future studies to address this issue.

In conclusion, in view of the low participation and high recall rates, it is crucial that breast cancer screening programs be based on performance indicators to optimize resource utilization and ensure the quality of the services provided. Moreover, a national framework and standardized performance indicators would mitigate this problem for other national programs that offer cancer screenings.
